# Zinc Oxide Nanoparticles Foliar Application Effectively Enhanced Zinc and Aroma Content in Rice (*Oryza sativa* L.) Grains

**DOI:** 10.1186/s12284-023-00653-0

**Published:** 2023-08-20

**Authors:** Rui Wang, Kailiang Mi, Xijun Yuan, Jie Chen, Jialing Pu, Xinyan Shi, Yanju Yang, Hongcheng Zhang, Haipeng Zhang

**Affiliations:** Innovation Center of Rice Cultivation Technology in Yangtze Valley, Ministry of Agriculture/Jiangsu Key Laboratory of Crop Cultivation and Physiology/Co-Innovation Center for Modern Production Technology of Grain Crops, Research Institute of Rice Industrial Engineering Technology, Yangzhou University, Yangzhou, 225009 China

**Keywords:** Zinc oxide nanoparticles, Foliar spraying, Zinc nutrition, Rice fragrance, Proline oxidase, 2-Acetyl-1-pyrroline

## Abstract

The search for an effective zinc fertilizer and its application method to effectively increase zinc content and enhance aroma in rice grains is a crucial objective. In this study, a 2-year field plot experiment was conducted to investigate the influence of ZnO NPs foliar spraying on rice quality, grain zinc and aroma content, along with exploring the physiological mechanisms underlying these effects. Our results demonstrated that the rice breakdown value and taste value of foliar spraying zinc oxide nanoparticles were improved by 31.0–41.7% and 8.2–13.0% compared with CK (control treatment involved spraying water), improving the tasting and steaming quality of rice. While Fe and Cu content in grains decreased for the application of zinc oxide nanoparticles, zinc oxide nanoparticles foliar spraying significantly increased the zinc content and accumulation of grains by 33.6–65.1% and 37.8–74.7%, respectively. Further analysis showed that the sprayed zinc oxide nanoparticles achieved effective enrichment of zinc in edible parts and increased the final bioavailability of Zn. In addition, foliar spraying of zinc oxide nanoparticles significantly increased activities of nitrate reductase and glutamine synthetase in leaves, which elevated nitrogen content in leaves and grains, and ultimately enhanced 2-acetyl-1-pyrroline (2-AP) content in grains at maturity by 6.1–21.4% compared to CK. Our findings indicated that zinc oxide nanoparticles can be practically applied as a foliar fertilizer at the gestation for quality improvement, zinc enrichment and aroma enhancement of rice grains.

## Introduction

Rice (*Oryza sativa* L.) is a crucial staple food crop globally, providing energy for around 70% of the world's population (Wang et al. 2018). The quality of rice plays a significant role in the daily life of individuals who consume rice as their primary food source. In recent years, due to social development and improved living standards, the demand for high-quality rice has increased, and consumers' preferences have shifted from high-yield to high-quality rice (Tang et al. 2019). Alongside conventional selection criteria for high-quality rice, such as ease of processing, good appearance, and excellent taste, rich nutrition and robust flavor have become essential factors for assessing rice quality by both the market and consumers. As a result, research on enhancing the nutritional value and aroma of rice has become a trending topic.

The aroma of rice is a complex mixture of various chemical compounds, and 2-acetyl-1-pyrroline (2-AP) is a critical odor-active component that serves as a representative index for measuring rice aroma in academic research (Bradbury et al. 2008; Prodhan and Qingyao 2020). The aroma of rice is not only influenced by internal genetic factors but also by external environmental conditions. Recent studies have found that the application of mineral elements such as zinc, selenium, and silicon can effectively increase the 2-AP content in rice (Luo et al. 2019). Additionally, the moderate application of mineral elements can enhance the mineral content in rice grains, improving the nutritional quality of rice and satisfying the human body's mineral requirements (Xu et al. 2022). Zinc is an essential mineral for human health, and its deficiency can cause various zinc deficiency disorders such as decreased immunity, delayed growth and development, and an increased risk of cancer (Nriagu. 2019). Since the human body cannot synthesize zinc, it must be obtained through external intake. However, compared to animal foods, the zinc content in plant-based foods like rice is lower (Rehman et al. 2017). In fact, the zinc content of rice is significantly lower than that of other plant foods such as wheat, corn, and potatoes (Cakmak et al. 2017). This suggests that long-term consumption of refined white rice may increase the risk of inadequate zinc intake. Therefore, the rational application of zinc fertilizer is crucial in improving the quality of rice, particularly in enhancing the zinc content and aroma of rice.

Numerous reports suggest that the moderate application of zinc fertilizer in Zn-deficient soil conditions can enhance rice yield, improve rice quality, and increase the zinc content in the grains (Saha et al. 2017). For instance, Camak et al. (2008) discovered that the application of zinc fertilizer significantly increased rice yield and zinc accumulation of grains in Zn-deficient soil. Kumar et al. (2017) and Zhang et al. (2021a) revealed that the incorporation of zinc fertilizer could improve rice processing quality, nutritional value, and cooking taste. Shivay et al. (2010) also observed that moderate zinc application positively influenced the water absorption and expansion of rice during cooking, leading to enhanced cooking quality. Moreover, Mo et al. (2016) found that the spraying of zinc fertilizer could considerably boost the activity of grain proline oxidase, which catalyzed the oxidation of proline to form the precursor substance of 2-AP (Huang et al. 2008). Hence, the application of zinc fertilizer through spraying can lead to an increase in the content of 2-AP in rice grains. A comparison between soil application and foliar spraying showed that the latter method is more effective in enhancing zinc content in grains as it bypasses the fixed absorption of zinc by soil and allows direct absorption by plants through leaf pores (Palmgren et al. 2008). Moreover, foliar spraying of zinc fertilizer did not significantly affect the phytic acid content in grains (Khampuang et al. 2020), which could prevent zinc from being bound by phytic acid and ultimately improve its bioavailability utilization (Rehman et al. 2012). Various new types of foliar zinc fertilizers have been developed and applied, including Zn-EDTA, which has shown higher absorption efficiency and enrichment effect in aromatic rice compared to other exogenous zinc (Naik and Das. 2007). Zinc glycine had also been found to have a better zinc-enriching effect than ZnSO_4_ and Zn-EDTA (Xu et al. 2022), it also had great zinc fortification and cadmium resistance (Lian et al. 2023). However, these new types of zinc fertilizers still have some drawbacks, such as low utilization rate, poor leaf nutrient attachment, high environmental impact, and potential for leaf burning (Ghasemi et al. 2013).

Compared to traditional inorganic zinc fertilizers such as ZnSO_4_ or ZnCl_2_, Zinc oxide nanoparticles (ZnO NPs) have been demonstrated to exhibit no specific toxicity and possess higher biocompatibility (Du et al. 2019b). Moreover, ZnO NPs are widely utilized in pesticides due to their excellent antimicrobial properties and stability (Raghunath and Perumal 2017; Abdelaziz et al. 2021). Owing to their large specific surface area and surface activity, ZnO NPs can be more effectively absorbed and utilized by crops, making them a promising new type of zinc fertilizer (Liu and Lal 2015). Bala et al. (2017) applied zinc oxide nanoparticles to rice seedlings grown in zinc-deficient soil and found that they effectively increased zinc content in the plants and changed soil microorganism populations and related enzyme activities. Applying ZnO NPs at the seedling stage also promoted rice growth and provide Zn supplementation (Afzal and Singh 2022). Additionally, ZnO NPs can be combined with NPK fertilizers to improve yield and fertilizer utilization rate (Giroto et al. 2022). Appropriate application of nano-zinc can significantly improve plant biomass, growth tolerance index, chlorophyll content, and antioxidant enzyme activity (Chutipaijit et al. 2018). The appropriate application of ZnO NPs has been demonstrated to be beneficial for rice growth and yield. However, the impact of ZnO NPs on rice quality, particularly regarding zinc nutrition and aroma of rice grains, remains unclear. In this study, we conducted foliar spraying of ZnO NPs at the gestation stage to investigate their effects on rice quality, grain zinc nutrition, and aroma content, with water spray used as the control. Additionally, we hypothesize that the application of ZnO NPs has a positive effect on grain aroma, and thus, we observed the dynamic changes of aroma synthesis-related substances at each stage from heading to maturity. This study aims to contribute to the practical application of ZnO NPs for improving rice quality, enhancing zinc enrichment, and promoting aroma development in grains. Moreover, we seek to elucidate the physiological mechanisms underlying the enhancement of grain aroma through ZnO NPs spray at the gestation stage.

## Materials and Methods

### Experimental Site

Experiments were carried out at the on-campus Experimental Farm of Yangzhou University (YZU), Jiangsu Province, P.R. China (32◦23.4′N, 119◦25.2′E) during the rice growing seasons of 2019 and 2020. The area is characterized by a subtropical monsoonal climate, featuring mild temperatures and ample rainfall (Yang et al. 2022). The average annual temperature is approximately 15.2 °C, with an annual precipitation of around 1020 mm (Wang et al. 2020). Meteorological data for the crop's growing season are provided in Table 1. The soil used in the experiment was sandy loam with 24.40 g kg^−1^ organic matter, 1.30 g kg^−1^ total nitrogen, 104.20 mg kg^−1^ available nitrogen, 35.40 mg kg^−1^ available phosphorus, and 72.50 mg kg^−1^ available potassium, with a soil pH of 6.51.

**Table 1:** Mean monthly temperatures and rainfall during the rice planting seasons

Month	Temperature (°C)		Rainfall (mm)	
	2019	2020	2019	2020
May	21.41	22.93	38.54	45.68
Jun	25.20	25.88	113.19	394.89
Jul	28.32	25.72	91.68	403.72
Aug	28.58	30.11	151.64	129.85
Sep	24.30	24.17	56.98	103.09
Oc	18.97	17.57	6.73	73.00

### Agronomic Management Systems

Nanjing 9108, a variety widely cultivated in the middle and lower reaches of the Yangtze River with a growth cycle of 150–155 days, was used as the research material. Seeds were sown on plastic nursery trays, and after a 48-h dark treatment, the seeds were the transferred to a moist nursery to allow for seedling growth. The 25-day-old seedlings were transplanted into the fields on June 12, 2019, and June 13, 2020, respectively. The seedlings were planted at a rate of 133.33 × 10^4^ seedlings per hectare, and were harvested on October 19, 2019, and October 20, 2020, respectively.

**Fig. 1:**
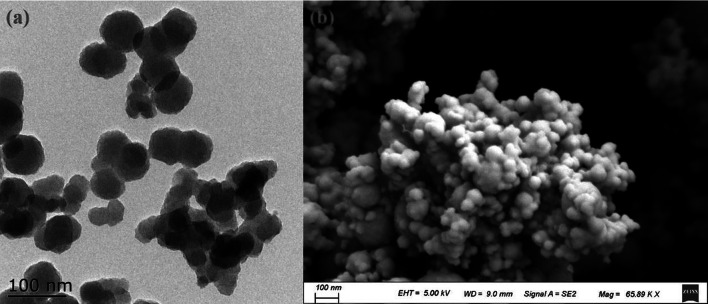
SEM (**a**) and TEM (**b**) images of ZnO NPs

This study utilized a randomized block design with 18 plots, each having dimensions of 10 m in length, 6 m in width, and an area of 60 square meters. The control treatment involved spraying water (CK), while five different dosages of nano-zinc were applied, including 0.75, 1.50, 3.00, 6.00, and 12.00 kg hm^−2^. Each treatment was replicated three times. The zinc oxide nanoparticles (ZnO NPs purity ≥ 99.9%) were purchased from Shanghai Chaowei Nanotechnology Co., Ltd (Shanghai, China). ZnO NPs used in the experiment were spherical particles with an average size of approximately 50 nm and a specific surface area of 133.6 m^2^ g^−1^, as confirmed by SEM and TEM characterization (refer to Fig. 1 for details). To prepare the treatment solutions, 4.5, 9.0, 18.0, 36.0, and 72.0 g of nanometer zinc were weighed and dissolved in 1 L of ultrapure water using ultrasound for 30 min. The solution was then sprayed on rice plants at the gestation stage on a clear and windless day. All the reagents used in the experiment were of analytical grade and were used without any further treatment before use.

The experiment employed a nitrogen application rate of 270 kg hm^−2^, with nitrogen being applied in three stages. During transplanting, 30% of nitrogen was applied, followed by 30% as tillering fertilizer seven days after transplantation, and the remaining 40% as panicle fertilizer. The N source used was urea, which contained 46% N. All the treatments were provided with phosphorus and potassium at 135 and 270 kg hm^−2^, respectively. Calcium super phosphate and potassium chloride were used as the basic fertilizer sources. Crop management practices such as irrigation, disease control, pest control, and weed control followed the recommended guidelines provided by local government agencies.

### Sampling and Data Collection

#### Yield and Yield Components

At maturity, rice from each plot was harvested and manually threshed, then sun-dried to a moisture level of approximately 14% to obtain the grain yield. The panicle number per hill was measured by counting the panicles of 20 rice plant hills from different locations in each plot. Furthermore, representative samples from 12 hills of rice plants were collected to measure and calculate the spikelet per panicle, seed-setting rate, and 1000-grain weight.

#### Rice Cooking Taste Quality

The rice taste meter (Satake Corporation, Higashi-Hiroshima, Japan) was employed to automatically measure the taste value of rice. The pasting properties of the rice flour were assessed using a rapid viscosity analyzer (RVA, Super3, Newport Scientific, Warriewood, Australia).

#### Determination of Trace Element Contents in Rice

The determination of trace element contents in rice was referred to the method of Momen et al. (2007) with some modification. In brief, 0.1 g of the dried sample was weighed and placed in an Abe bottle. The bottle was then placed in a muffle furnace and ashed at 480 °C for approximately 14 h. Once cooled, 2 mL of 15% HNO_3_ solution was added to the bottle, which was allowed to stand for 24 h. Then, 8 mL of ultra-pure water was added, and the solution was filtered using slow speed filter paper. The resulting filtrate was diluted, and the trace element content was determined using a plasma emission spectrometry-atomic absorption spectrometer (iCAP 6300, Thermo Fisher, Waltham, USA).

#### Determination of the Phytic Acid Content and Molar Ratio of Phytic Acid to Zn

For determining the phytic acid content, a modified version of the Vaintraub and Lapteva (1988) method was employed. Firstly, 0.25 g of the dried sample was mixed with 5 ml of 0.7% diluted hydrochloric acid, shaken at 25 °C and 150 r min^−1^ for 1 h, and then centrifuged at 4000 r min^−1^ for 15 min to obtain a supernatant. Next, 0.5 ml of the supernatant was aspirated and mixed with 2.5 ml of deionized water. The resulting mixture was then combined with 1 ml of chromogenic agents (0.03% FeCl_3_ and 0.3% sulfosalicylic acid), along with a standard solution prepared using sodium phytate.

Afterward, the absorbance value at 500 nm was measured using an ultraviolet spectrophotometer (G-9, Feller Instrument Co., Ltd, Nanjing, China). A standard solution was prepared from sodium phytate (C_6_H_8_Na_12_O_24_P_6_ purity ≥ 99.9%, Wokai Biotechnology Co., Beijing, China), and a standard curve was constructed using the absorbance as the vertical coordinate and the concentration of the standard solution as the horizontal coordinate. The concentration of phytic acid in the sample was then calculated based on the absorbance value using the following specific formula:

1 $$c = {{\left[ {\frac{{(y - y_{b} - b)}}{a} \times \frac{{v_{1} }}{{v_{2} }}} \right]} \mathord{\left/ {\vphantom {{\left[ {\frac{{(y - y_{b} - b)}}{a} \times \frac{{v_{1} }}{{v_{2} }}} \right]} m}} \right. \kern-0pt} m}$$

where* c* refers to the concentration of phytic acid in the sample, *y* refers to the absorbance, *y*_*b*_ refers to the absorbance of the same treatment without sample addition, *v*_1_ refers to the volume of the supernatant, *v*_2_ refers to the the volume of supernatant aspirated, *m* refers to the mass of the sample. The molar ratio of phytic acid to zinc was determined by dividing the millimoles of phytic acid by the millimoles of zinc.

#### Determination of the Activity of Nitrate Reductase and Glutamine Synthetase

The determination of nitrate reductase activity (NRA) in rice was referred to the method of Xi et al. (2020) with some modification. To extract the original solution from the fresh leaf samples, 0.1 g was weighed and ground in a tissue grinder (MM400, Retsch, Haan, Germany) under liquid nitrogen conditions. After complete crushing, 1 ml of extraction medium (0.1 mol L^−1^, pH 7.5; containing 12% 1-propanol and 0.1 mol L^−1^ KNO_3_) was added to the sample. A separate control sample was prepared using KNO_3_-free phosphate buffer. All tubes were placed in a desiccator, evacuated by a hollow pump for 2 min, and then incubated in a 30 °C water bath for 30 min. After the incubation, 1 ml of reaction solution was taken from each tube, followed by the addition of 1 ml of sulfanilamide solution to stop the enzyme reaction. Subsequently, 1 ml of α-naphthylamine solution was added, and the color was allowed to develop for 15 min. Afterward, the tubes were centrifuged at 3000 r min^−1^ for 10 min, and the supernatant was collected for absorbance measurement at 540 nm using an enzyme marker (Infinite M200 Pro, Tecan, Männedorf, Switzerland). The standard solution used for calibration was prepared from sodium nitrite.

Glutamine synthetase activity (GSA) in rice was determined following the method of Luo et al. (2014) with slight modifications. Fresh leaf samples (0.1 g) were ground in a ball mill, and then 1 ml of extraction buffer (0.05 mol L^−1^ Tris–HCl, pH 8.0; containing 2 mmol L^−1^ Mg^2+^, 2 mmol L^−1^ DTT, and 0.4 mol L^−1^ sucrose) was added. After homogenization, the mixture was transferred to a 5 ml centrifuge tube and centrifuged at 8000 r min^−1^ for 20 min at 4 °C to collect the supernatant. To 0.7 ml of the supernatant, 0.7 ml of ATP solution (40 mmol L^−1^) was added, and then combined with 1.6 ml of reaction solution A (0.1 mol L^−1^ Tris–HCl, pH 7.4; 80 mmol L^−1^ Mg^2+^, 20 mmol L^−1^ monosodium glutamate, 20 mmol L^−1^ cysteine, 2 mmol L^−1^ EGTA, and 80 mmol L^−1^ hydroxylamine hydrochloride). The mixture was incubated at 37 °C for 30 min. Next, 1 ml of colorant (0.2 mol L^−1^ TCA, 0.37 mol L^−1^ FeCl_3_, and 0.6 mol L^−1^ HCl) was added, and after shaking and standing for a moment, the mixture was centrifuged at 5000 r min^−1^ for 10 min. The supernatant was used for absorbance measurement at 540 nm using an enzyme marker (Infinite M200 Pro, Tecan, Männedorf, Switzerland), with 1.6 ml of reaction mixture B (0.1 mol L^−1^ Tris–HCl, pH 7.4; 80 mmol L^−1^ Mg^2+^, 20 mmol L^−1^ monosodium glutamate, 20 mmol L^−1^ cysteine, 2 mmol L^−1^ EGTA) used as the control. A 0.5 ml aliquot of the supernatant was diluted to 100 ml with pure water for soluble protein determination using Caulmers Brilliant Blue G-250, with 2 ml of the supernatant used for this purpose. All the reagents for NRA and GSA assays were obtained from Keming Biotechnology Co., Ltd (Suzhou, China).

#### Determination of Nitrogen Content of Rice

At heading, 10 days and 25 days after heading, and maturity stage, 20 rice plants were continuously taken from each block. Only the rice leaves and panicle were taken off. After drying to constant weight in the oven at 70 °C, the plants were ground and digested, and the N content of each rice part was measured by Kjeldahl method (Bremner. 2009).

#### Determination of Proline and Proline Oxidase Activity

At the heading stage, 10, 25 days after heading, and at the maturity stage, fresh rice grain samples were collected and immediately frozen in liquid nitrogen. The frozen samples were then ground to a fine powder using a mixed ball mill (MM400, Retsch, Haan, German) and stored in a refrigerator at − 70 °C until further analysis.

The extraction and determination of proline followed the procedure outlined by Du et al. (2019a), with some modifications. Rice grain powder was mixed with 3% sulfosalicylic acid and homogenized in a boiling water bath. The resulting extract was heated and reacted with acidic ninhydrin before being extracted with toluene. The toluene extraction was then analyzed for the absorbance of the red chromophore at 520 nm.

The activity of proline oxidase was measured with modifications to the procedure outlined by Yang et al. (2022). First, 0.1 g of fresh rice grain samples were ground with a tissue grinder (MM400, Retsch, Haan, German) under liquid nitrogen conditions. After complete crushing, 2 ml of extraction medium (0.1 mol L^−1^ potassium phosphate buffer, pH 7.4; 0.5% Triton X-100) was added to obtain a homogenate. The homogenate was then transferred to a 5 ml centrifuge tube, and the proline oxidase solution was obtained by centrifugation at 3000 r min^−1^ at 4 °C for 10 min. Next, 0.2 ml of the enzyme solution was aspirated and added to 0.3 ml of the reaction system (15 mmol L^−1^ L-Proline; 0.01 mol L^−1^ Cytochrome C; 0.1 mol L^−1^ potassium phosphate buffer, pH 8.0; 0.5% Triton X-100). After incubation at 37 °C for 30 min, the reaction was terminated by adding 1 ml of 10% TCA. Then, 0.3 ml of 0.5% 2-aminobenzaldehyde (dissolved in 95% ethanol) was added, and the mixture was allowed to react for 10 min. Finally, the mixture was centrifuged at 9000 r min^−1^ for 10 min, and the absorbance was measured at 440 nm. All the reagents for the activity of proline oxidase assays were obtained from Keming Biotechnology Co., Ltd (Suzhou, China).

#### Determination of 2-Ap Content in Rice Grain

The 2-acetyl-1-pyrroline (2-AP) content was determined by following the methods described by Yang et al. (2022) with some modifications. Fresh grain samples were subjected to ultrasound-assisted solvent extraction (UASE) using chromatographically pure dichloromethane to separate the 2-AP. To the headspace vial, 2,4,6-collidine was added as an internal standard, and the 2-AP content was analyzed using GC–MS (GCMS-8890A+5977B, Agilent, USA) immediately. The content of 2-AP was expressed as μg g^−1^ of fresh weight (FW).

#### Statistical Analysis

The data were presented as mean ± standard deviation (SD), and statistical analyses were conducted using IBM SPSS Statistics software (Version 26.0, USA). One-way analysis of variance (ANOVA) was performed to identify significant differences (*p* < 0.05) among treatment means.

## Results

### Yield and Yield Components

Table 2 displays the impact of ZnO NPs on rice yield and its components. Application of ZnO NPs through foliar spraying resulted in a significant increase in grain yield by 2.3% to 4.1% in comparison to the control (CK). During the two-year study, the augmented yield of rice plants treated with ZnO NPs spraying could be attributed to the increase in the number of spikelets per spike (7.4% to 9.2%), filled grain rate (1.7% to 4.3%), and 1000-grain weight (4.2% to 7.1%). Nevertheless, the ANOVA results revealed that the variation in panicle number was not significantly affected by nanoscale zinc spraying.

**Table 2:** Effects of foliar spraying ZnO NPs on rice yield and its components

Year	Treatment	Panicles (× 10^6^ hm^−2^)	Spikelets per panicle	Filled grain rate (%)	1000-Grain weight (g)	Grain yield (t hm^−2^)
2019	CK	3.54 ± 0.20a	110.76 ± 8.52b	90.33 ± 0.60c	25.33 ± 0.55b	9.84 ± 0.20b
	T1	3.56 ± 0.26a	119.07 ± 3.14ab	91.30 ± 1.07c	26.30 ± 0.90ab	10.09 ± 0.11ab
	T2	3.55 ± 0.20a	119.30 ± 4.02ab	92.35 ± 1.70bc	26.63 ± 0.19a	10.14 ± 0.04a
	T3	3.54 ± 0.20a	119.37 ± 3.39ab	92.47 ± 0.57bc	26.73 ± 0.33a	10.19 ± 0.06a
	T4	3.58 ± 0.07a	119.63 ± 6.18ab	93.49 ± 0.75ab	27.05 ± 0.11a	10.22 ± 0.19a
	T5	3.55 ± 0.33a	120.92 ± 2.72a	94.94 ± 0.43a	27.08 ± 0.24a	10.26 ± 0.13a
2020	CK	3.52 ± 0.07a	107.82 ± 1.50b	89.95 ± 0.35b	25.48 ± 0.20b	9.97 ± 0.10b
	T1	3.53 ± 0.14a	115.63 ± 0.88ab	92.09 ± 1.42ab	26.62 ± 0.23ab	10.18 ± 0.15a
	T2	3.56 ± 0.18a	115.69 ± 2.03ab	92.25 ± 0.25ab	26.86 ± 0.50ab	10.23 ± 0.08a
	T3	3.55 ± 0.25a	115.74 ± 6.10ab	92.47 ± 0.86a	27.20 ± 0.51a	10.29 ± 0.04a
	T4	3.52 ± 0.29a	117.04 ± 4.50a	92.49 ± 1.39a	27.24 ± 0.19a	10.31 ± 0.13a
	T5	3.61 ± 0.26a	117.70 ± 1.96a	93.04 ± 2.01a	27.32 ± 1.75a	10.36 ± 0.04a

Values within the same column followed by different letters are significantly different at the 0.05 probability level

### Rice Quality

Table 3 and Table 4 present the impact of foliar spraying ZnO NPs on the steaming and tasting quality of rice. The breakdown values of rice treated with ZnO NPs showed a significant increase of 31.0–41.7% compared to the control (CK), with the highest values observed at T4, and the trend varied with the dosage of sprayed nano Zn. On the other hand, the setback values of rice treated with ZnO NPs decreased by 71.9–106.4% compared to CK, and significant reductions were evident in treatments T2–T5. Further analysis indicated that the increase in breakdown value and decrease in setback value were primarily due to a significant reduction in rice viscosity. Moreover, the foliar spraying of ZnO NPs had significant positive effects on rice appearance value, viscosity value, and balance value, while showing a negative impact on hardness value. Overall, the taste value of rice treated with ZnO NPs improved by 7.7–13.0% compared to CK, with significant enhancements observed in treatments T2–T5, and the trend of rice taste values was consistent with the disintegration values.

**Table 3:** Effects of foliar spraying ZnO NPs on RVA parameters of rice

Year	Treatment	Peak viscosity (cP)	Trough viscosity (cP)	Final viscosity (cP)	Breakdown (cP)	Setback (cP)
2019	CK	2655.00 ± 32.14b	2004.67 ± 97.68a	2478.00 ± 91.91a	650.33 ± 61.34b	− 177.00 ± 20.06a
	T1	2725.67 ± 14.57a	1846.00 ± 55.47ab	2388.67 ± 38.68a	879.67 ± 43.53a	− 337.00 ± 25.82ab
	T2	2742.00 ± 27.50a	1758.67 ± 74.81ab	2313.33 ± 60.48a	983.33 ± 74.77a	− 428.67 ± 63.57b
	T3	2747.00 ± 18.52a	1753.33 ± 11.59b	2386.33 ± 14.74a	993.67 ± 27.65a	− 360.67 ± 11.24ab
	T4	2696.00 ± 41.94ab	1712.67 ± 22.55b	2351.33 ± 41.04a	983.33 ± 20.43a	− 344.67 ± 30.01ab
	T5	2727.33 ± 17.79a	1830.33 ± 69.74ab	2358.33 ± 80.16a	897.00 ± 47.22a	− 369.00 ± 62.45b
2020	CK	2708.00 ± 42.58ab	1937.33 ± 64.85a	2430.67 ± 154.05a	770.67 ± 36.26b	− 277.33 ± 28.00a
	T1	2683.33 ± 15.95ab	1706.33 ± 28.57b	2239.33 ± 35.57b	977.00 ± 37.27a	− 444.00 ± 44.03b
	T2	2602.67 ± 10.02c	1604.33 ± 51.16bc	2168.00 ± 50.69bc	998.33 ± 60.93a	− 434.67 ± 60.01b
	T3	2661.33 ± 14.47b	1652.00 ± 42.00bc	2235.67 ± 32.25bc	1009.33 ± 40.02a	− 425.67 ± 20.40b
	T4	2732.67 ± 36.35a	1714.33 ± 43.36b	2259.33 ± 11.37b	1018.33 ± 52.37a	− 473.33 ± 32.03b
	T5	2559.67 ± 12.52c	1544.00 ± 29.51c	2118.67 ± 34.79c	1015.67 ± 31.47a	− 441.00 ± 37.04b

Values within the same column followed by different letters are significantly different at the 0.05 probability level

**Table 4:** Effects of foliar spraying ZnO NPs on taste qualities of rice

Year	Treatment	Taste value	Appearance value	Hardness value	Viscosity value	Balance value
2019	CK	71.27 ± 1.68b	6.67 ± 0.26b	6.70 ± 0.14ab	7.20 ± 0.22b	6.70 ± 0.28b
	T1	78.17 ± 0.63ab	7.77 ± 0.12a	6.20 ± 0.08b	8.43 ± 0.17a	7.87 ± 0.12a
	T2	79.17 ± 1.53a	7.97 ± 0.24a	6.37 ± 0.05ab	8.43 ± 0.21a	8.03 ± 0.26a
	T3	78.97 ± 1.91a	7.87 ± 0.29a	6.07 ± 0.24b	8.57 ± 0.21a	7.97 ± 0.29a
	T4	82.37 ± 2.22a	8.07 ± 1.01a	6.03 ± 0.79b	8.60 ± 0.86a	8.13 ± 0.97a
	T5	79.03 ± 2.95a	7.97 ± 0.41a	7.00 ± 0.17a	8.37 ± 0.33a	8.03 ± 0.42a
2020	CK	73.97 ± 1.41b	7.40 ± 0.16b	6.27 ± 0.51ab	7.50 ± 0.24c	7.33 ± 0.12b
	T1	79.00 ± 2.16ab	7.93 ± 0.31ab	6.03 ± 0.19b	8.40 ± 0.22b	8.03 ± 0.31a
	T2	79.33 ± 2.62a	8.00 ± 0.36ab	6.07 ± 0.12b	8.47 ± 0.31ab	8.10 ± 0.36a
	T3	80.67 ± 2.05a	8.17 ± 0.34a	6.00 ± 0.14b	8.63 ± 0.12ab	8.30 ± 0.29a
	T4	81.67 ± 1.70a	8.33 ± 0.21a	5.90 ± 0.08b	8.70 ± 0.14ab	8.43 ± 0.16a
	T5	81.33 ± 2.62a	8.23 ± 0.26a	6.80 ± 0.51a	9.17 ± 0.58a	8.30 ± 0.29a

Values within the same column followed by different letters are significantly different at the 0.05 probability level

As presented in Table 5, the foliar spray of ZnO NPs had no significant effect on the content of Mn and Se in rice grains. While in treatment T1, ZnO NPs spray slightly increased the uptake of Fe and Cu, but the effect was not significant. However, with the increase in the dosage of ZnO NPs application, the foliar spray at gestation generally showed a negative impact on the uptake of Fe and Cu in rice grains. There was a significant decrease by 2.2–6.7% and 3.7–10.8% in Fe and Cu content, respectively, in treatments T2–T5.

**Table 5:** Effects of foliar spraying ZnO NPs on trace element concentrations of rice

Year	Treatment	Fe (mg kg^−1^)	Mn (mg kg^−1^)	Cu (mg kg^−1^)	Se (mg kg^−1^)
2019	CK	17.19 ± 0.09a	24.98 ± 0.27ab	6.22 ± 0.07a	2.11 ± 0.04a
	T1	17.33 ± 0.17a	25.10 ± 0.24a	6.27 ± 0.05a	2.15 ± 0.07a
	T2	16.24 ± 0.11d	24.94 ± 0.26ab	5.69 ± 0.08 cd	2.21 ± 0.10a
	T3	16.93 ± 0.21b	24.74 ± 0.19b	5.80 ± 0.11c	2.14 ± 0.06a
	T4	16.01 ± 0.16e	25.00 ± 0.38ab	6.08 ± 0.04b	2.11 ± 0.06a
	T5	16.55 ± 0.17c	25.02 ± 0.36ab	5.58 ± 0.08d	2.13 ± 0.04a
2020	CK	17.23 ± 0.17a	24.97 ± 0.31a	6.26 ± 0.10a	2.18 ± 0.03a
	T1	17.35 ± 0.13a	25.05 ± 0.23a	6.33 ± 0.05a	2.19 ± 0.05a
	T2	16.89 ± 0.19b	24.88 ± 0.13a	5.63 ± 0.05d	2.22 ± 0.04a
	T3	16.10 ± 0.11d	24.93 ± 0.22a	6.10 ± 0.03b	2.22 ± 0.05a
	T4	17.00 ± 0.15b	25.03 ± 0.20a	5.71 ± 0.03d	2.24 ± 0.11a
	T5	16.43 ± 0.24c	24.93 ± 0.28a	5.89 ± 0.04c	2.18 ± 0.03a

Values within the same column followed by different letters are significantly different at the 0.05 probability level

### Zn Content and Accumulation in Rice Grains

**Fig. 2:**
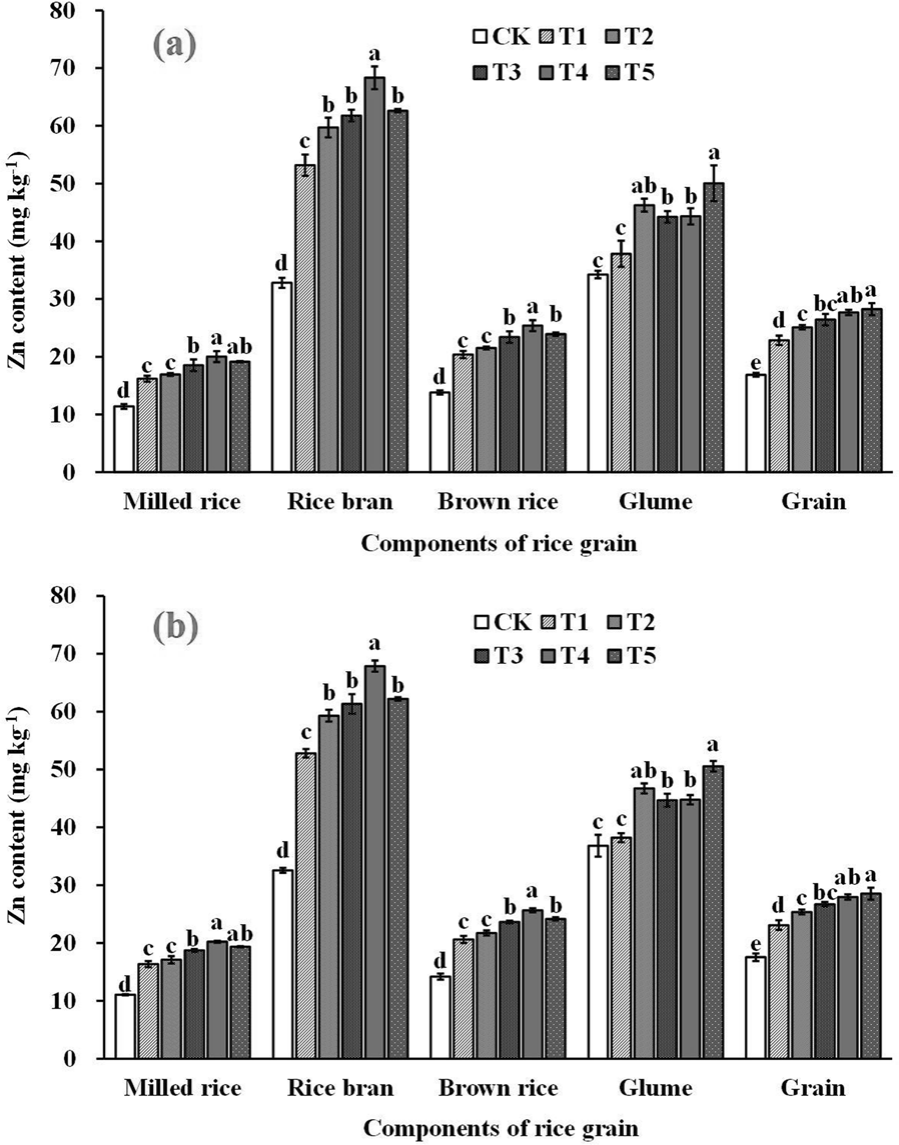
Zn content in rice grains in 2019 (**a**) and 2020 (**b**). Error bars show standard error of replicates (n = 3). Values followed by different lowercase letters were significantly different at the 0.05 probability level among different treatments

**Fig. 3:**
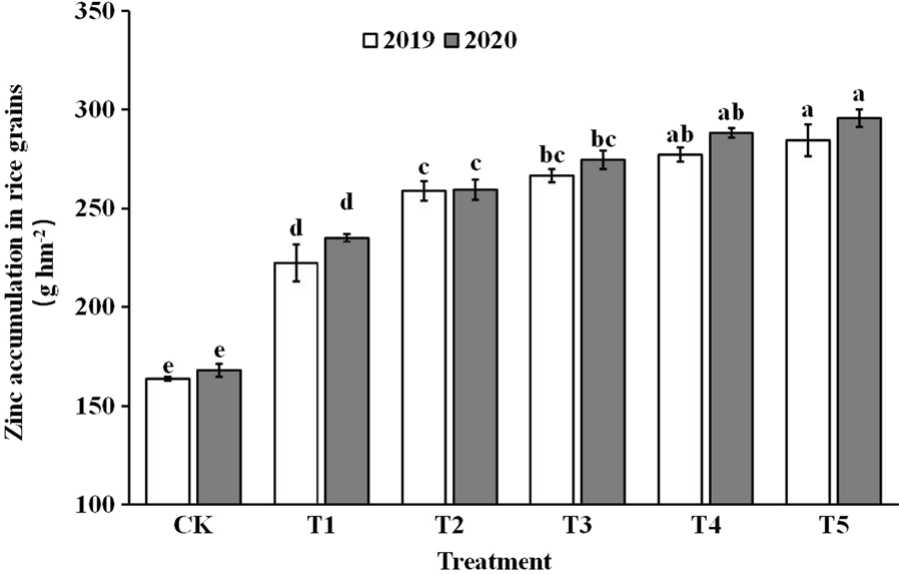
Zn accumulation in rice grains. Error bars show standard error of replicates (n = 3). Values followed by different lowercase letters were significantly different at the 0.05 probability level among different treatments

Figure 2 and Fig. 3 shows that foliar spraying of ZnO NPs had a significant impact on the Zn content and accumulation in rice grains, resulting in an increase of 33.6–65.1% and 37.8–74.7%, respectively, compared to the control. The Zn content in brown rice and glume treated with foliar sprays of ZnO NPs at the gestation stage increased by 46.4–82.4% and 7.1–41.7%, respectively. For edible fine rice and inedible rice bran, spraying of ZnO NPs increased Zn content by 45.1–79.4% and 62.1–108.4%. The Zn content in brown rice, rice bran, and milled rice increased and then decreased with increasing application of ZnO NP levels, reaching the highest value in the T4. The effect of ZnO NPs dosage on the Zn content in brown rice was slightly inferior to that in the grains and rice glume.

### Zn Distribution in Rice Grains

**Fig. 4:**
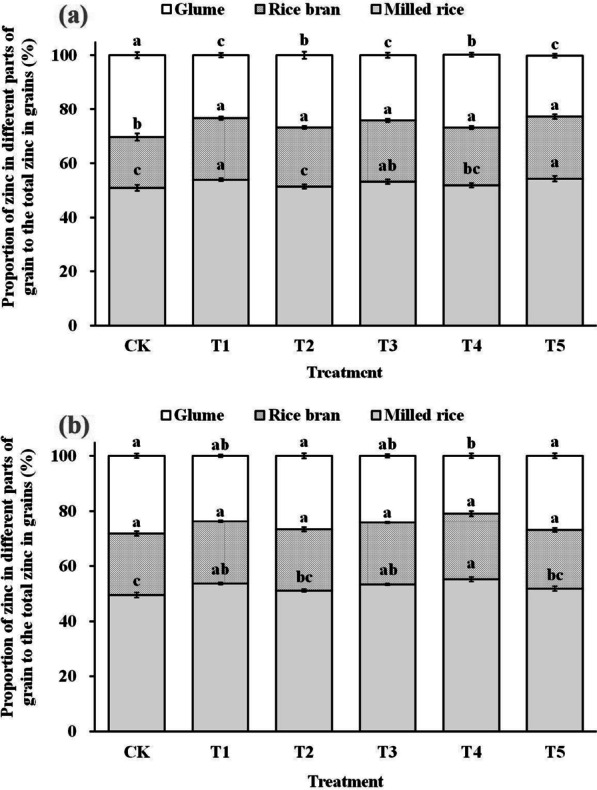
The distribution of zinc accumulation in each part of the grain relative to the total zinc content in the grain in 2019 (**a**) and 2020 (**b**). Error bars show standard error of replicates (n = 3). Values followed by different lowercase letters were significantly different at the 0.05 probability level among different treatments

Additional analysis of the distribution of zinc accumulation in each part of the grain relative to the total zinc content in the grain indicated that foliar spraying of ZnO NPs increased the proportion of zinc in the edible parts such as brown rice and polished rice compared to the total zinc content in the grain, while decreasing the proportion of non-edible parts such as glume and rice bran relative to the total zinc content in the grain (Fig. 4).

### The Phytic Acid to Zinc Molar Ratio

**Fig. 5:**
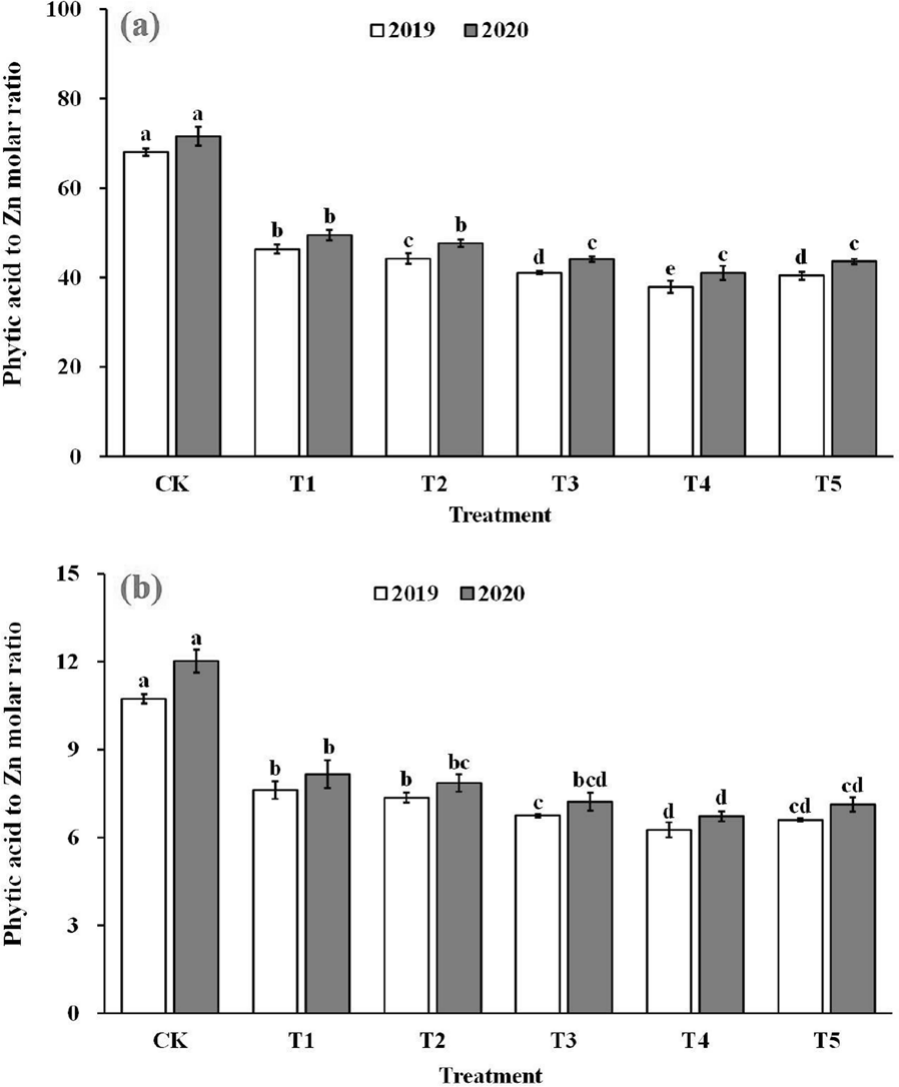
The phytic acid to zinc molar ratio in brown rice (**a**) and polished rice (**b**). Error bars show standard error of replicates (n = 3). Values followed by different lowercase letters were significantly different at the 0.05 probability level among different treatments

The improvement in the bioavailability of zinc by spraying ZnO NPs during gestation was also reflected in the molar ratio of phytate to zinc in brown and polished rice (Fig. 5). The phytic acid to zinc molar ratio in brown rice and polished rice significantly decreased by 31.4–43.5% and 30.6–42.9% when treated with ZnO NPs. The phytic acid to zinc molar ratio in brown rice and polished rice decreased and then increased with increasing application of ZnO NP levels, reaching the lowest value in the T4.

### The Activities of NR and GS of Flag Leaf After Heading

**Fig. 6:**
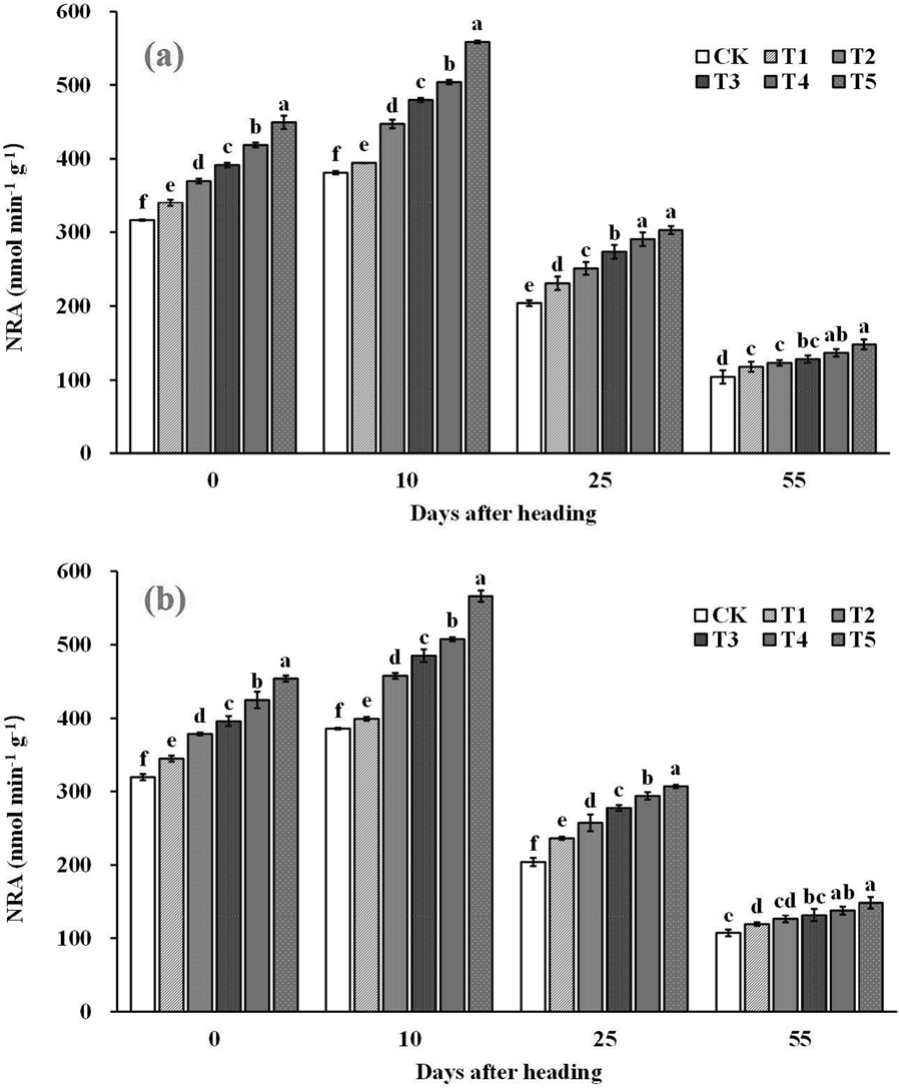
Dynamic changes of nitrate reductase activity (NRA) in rice leaves after heading in 2019 (**a**) and 2020 (**b**). Error bars show standard error of replicates (n = 3). Values followed by different lowercase letters were significantly different at the 0.05 probability level among different treatments

**Fig. 7:**
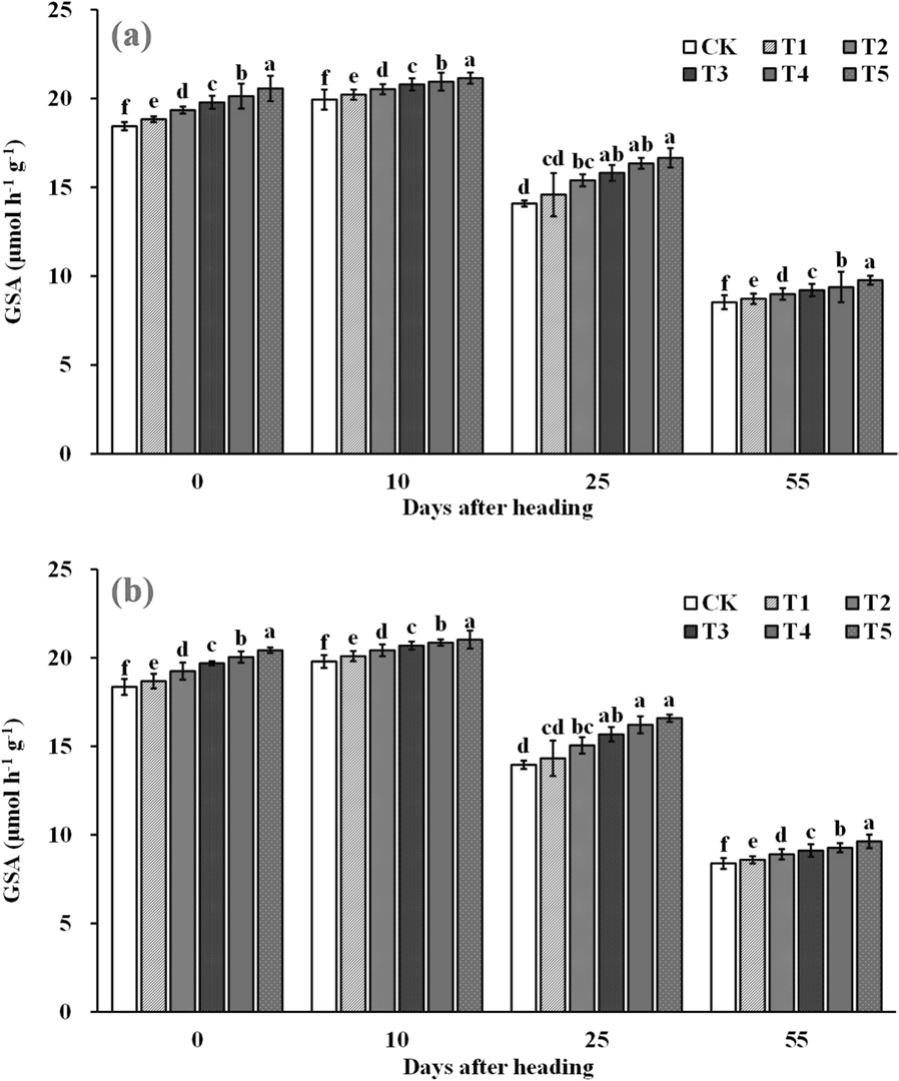
Dynamic changes of glutamine synthetase activity (GSA) in rice leaves after heading in 2019 (**a**) and 2020 (**b**). Error bars show standard error of replicates (n = 3). Values followed by different lowercase letters were significantly different at the 0.05 probability level among different treatments

The results in Fig. 6 and Fig. 7 show that both NRA and GSA in the leaves of all treatments increased and then decreased with time from heading to maturity stage, peaking at 10 days after heading. Spraying ZnO NPs significantly increased NRA and GSA at heading stage, 10 days after heading, 25 days after heading, and maturity stage. Compared to the control (CK), NRA with spraying ZnO NPs at heading stage, 10 days after heading, 25 days after heading, and maturity stage was significantly increased by 7.7–42.1%, 3.6–46.7%, 14.5–49.4%, and 12.2–40.7%, respectively. As for GSA, compared with the CK, GSA at heading stage, 10 days after heading, 25 days after heading, and mature stage was significantly increased by 1.9–11.4%, 1.3–6.1%, 3.1–18.5%, and 2.3–14.8%, respectively.

### The Nitrogen Content in Leaves and Grains After Heading

**Fig. 8:**
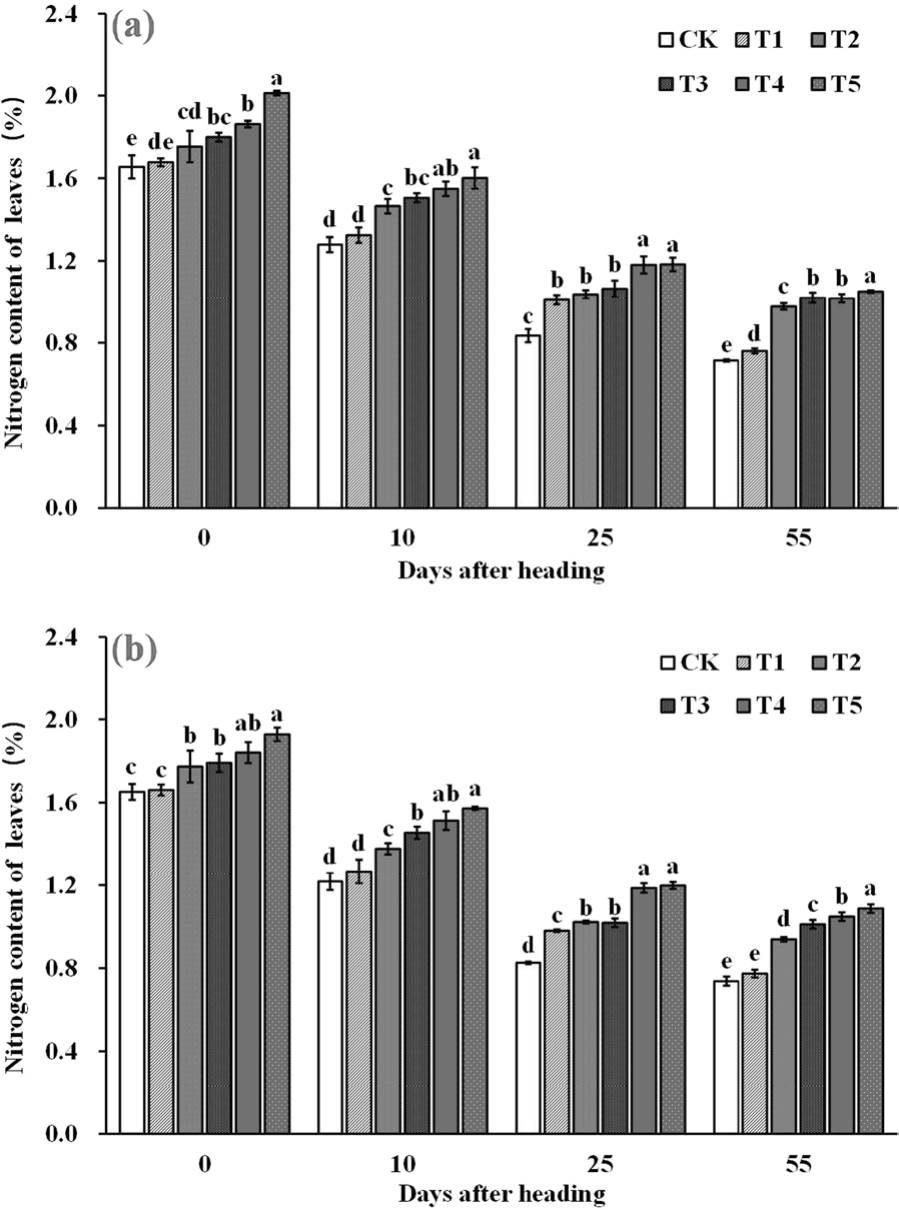
Dynamic changes of nitrogen content of leaves after heading in 2019 (**a**) and 2020 (**b**). Error bars show standard error of replicates (n = 3). Values followed by different lowercase letters were significantly different at the 0.05 probability level among different treatments

The application of ZnO NPs resulted in a significant increase in leaves N content (Fig. 8). Leaves N content increased with the increase of ZnO NPs spraying. Compared to CK, T2–T5 significantly increased leaves N content by 10.7–23.5%, 12.2–24.2%, 13.4–24.7% and 12.1–23.7% at the heading stage, 10 days after heading, 25 days after heading, and maturity, respectively. Moreover, the improvement effect of ZnO NPs spraying on leaves N content was more significant at 25 days after heading and maturity, with T5 treatment showing the most remarkable results.

**Fig. 9:**
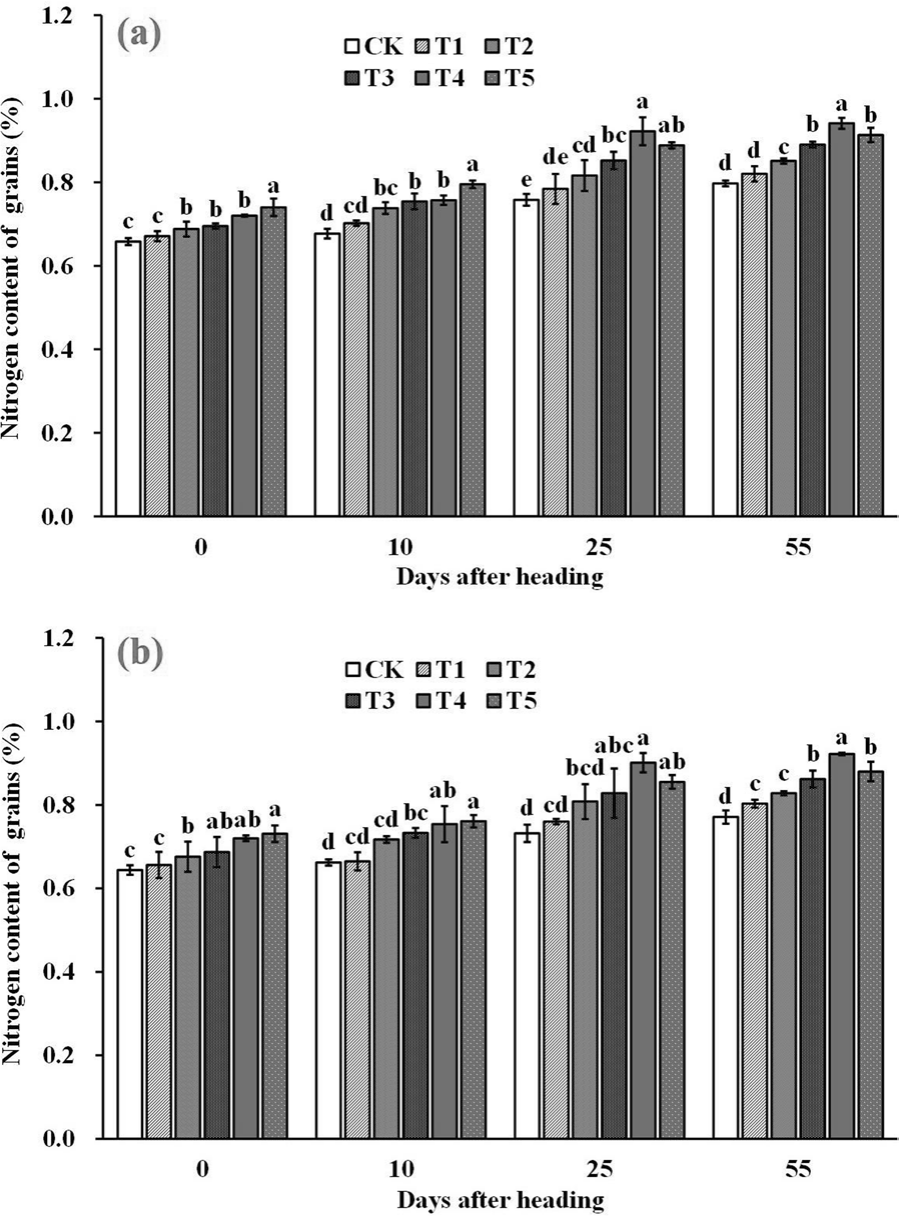
Dynamic changes of nitrogen content of grains after heading in 2019 (**a**) and 2020 (**b**). Error bars show standard error of replicates (n = 3). Values followed by different lowercase letters were significantly different at the 0.05 probability level among different treatments

The nitrogen content of grains was also affected by the spraying of ZnO NPs (Fig. 9). At the heading stage and 10 days after heading, the nitrogen content of grains increased with increasing ZnO NPs dosage. However, at 25 days after heading and maturity stage, the nitrogen content of grains increased first and then decreased with increasing ZnO NPs dosage, reaching the highest value at T4. Compared to the control (CK), spraying ZnO NPs significantly increased grain nitrogen content by 6.9–13.1% under T3–T5 treatment at heading stage, 9.0–16.4% under T2–T5 treatment at 10 days after heading, and 12.8–22.2% under T3–T5 treatment at 25 days after heading. At maturity, T2–T5 treatment significantly increased grain nitrogen content by 7.0–18.5%.

### The Proline Content in Grains After Heading

**Fig. 10:**
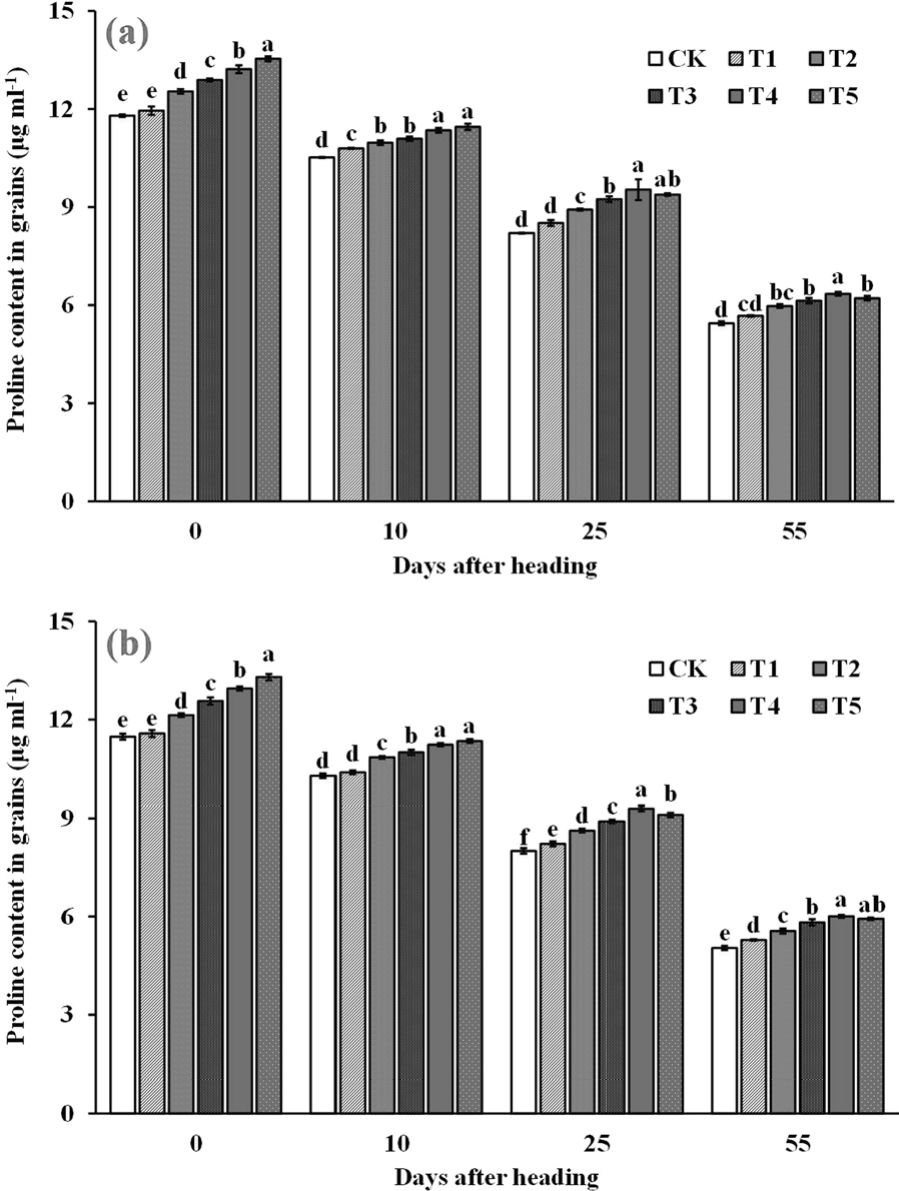
Dynamic changes of proline content of grains after heading in 2019 (**a**) and 2020 (**b**). Error bars show standard error of replicates (n = 3). Values followed by different lowercase letters were significantly different at the 0.05 probability level among different treatments

As shown in Fig. 10, the grain proline content decreased gradually with time after rice heading, but spraying ZnO NPs increased the proline content at all stages after heading. The results of the two-year experiment showed that the proline content in the grains increased with the amount of ZnO NPs sprayed at the heading stage and 10 days after heading. Compared to the control group (CK), the proline content increased significantly by an average of 6.0–15.3% at T2–T5 of the heading stage and by 4.9–9.6% at T2–T5 of 10 days after heading for the ZnO NPs sprayed treatment over the two years. However, the proline content in the grains increased and then decreased with increasing spraying of ZnO NPs at day 25 after heading and at maturity, reaching the highest level at T4. In the two-year trial, the proline content in grains sprayed with ZnO NPs significantly increased by 3.1–16.1% and 3.6–17.9% compared to CK at 25 days after heading and at maturity.

### The Proline Oxidase Activity in Grains After Heading

**Fig. 11:**
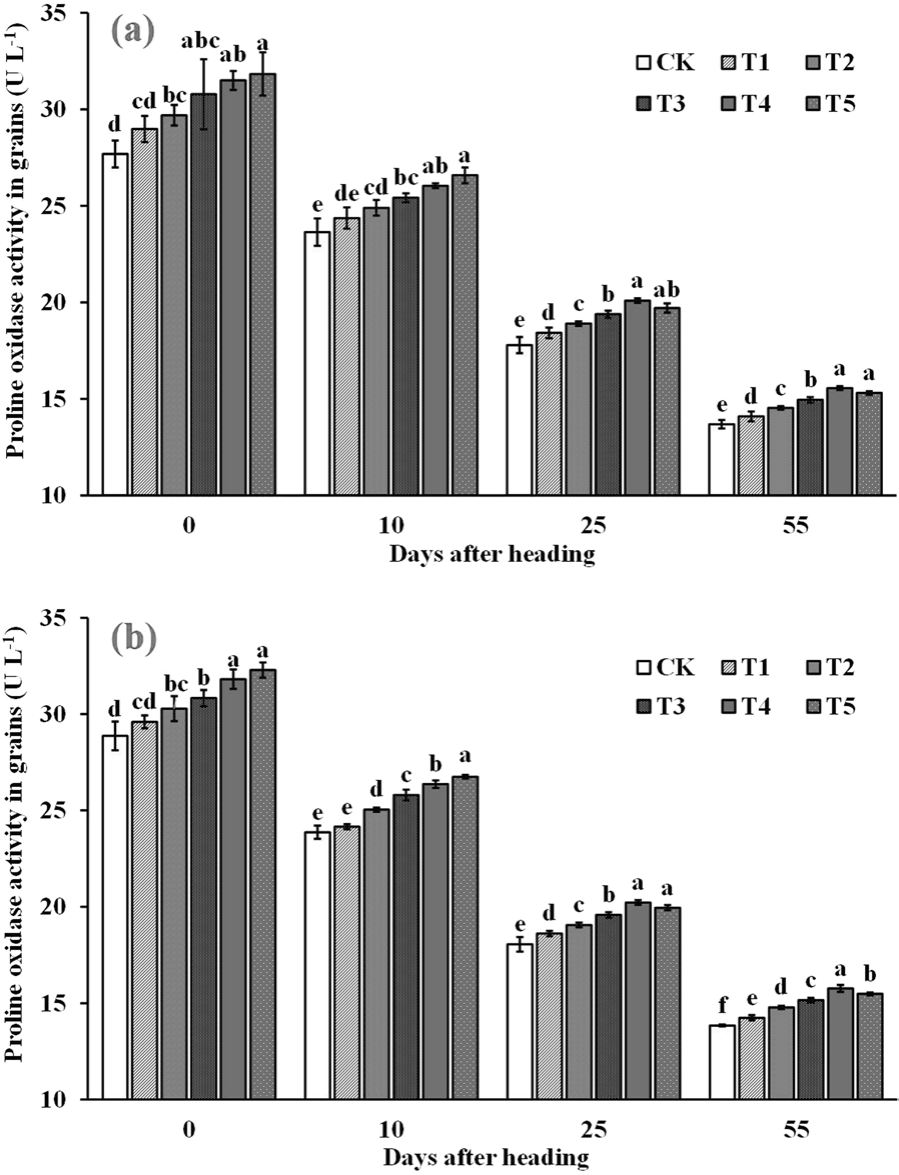
Dynamic changes of proline oxidase of grains after heading in 2019 (**a**) and 2020 (**b**). Error bars show standard error of replicates (n = 3). Values followed by different lowercase letters were significantly different at the 0.05 probability level among different treatments

The trend of proline oxidase activity in rice grains was consistent with that of grains after heading, as indicated in Fig. 11. Spraying ZnO NPs significantly improved the proline oxidase activity in grains during the heading-maturity stage. The proline oxidase activity of grains in the treatment of spraying ZnO NPs increased with the increase of the amount of nano zinc at the heading stage and 10 days after heading. Except for the T1, the effect of spraying ZnO NPs on the proline activity of grains reached a significant level compared to CK. However, on the 25th day after spike and at the maturity stage, the activity of proline oxidase in grains increased first and then decreased with the increase of ZnO NPs dosage, reaching the highest level at T4. At 25 days after heading, the proline oxidase activity of grains sprayed with nano zinc was significantly increased by 3.3–12.4% compared to CK. At the mature stage, the proline oxidase activity of T2–T5 significantly increased by 2.9–13.8% compared to spraying water.

### The 2-AP Content in Grains After Heading

**Fig. 12:**
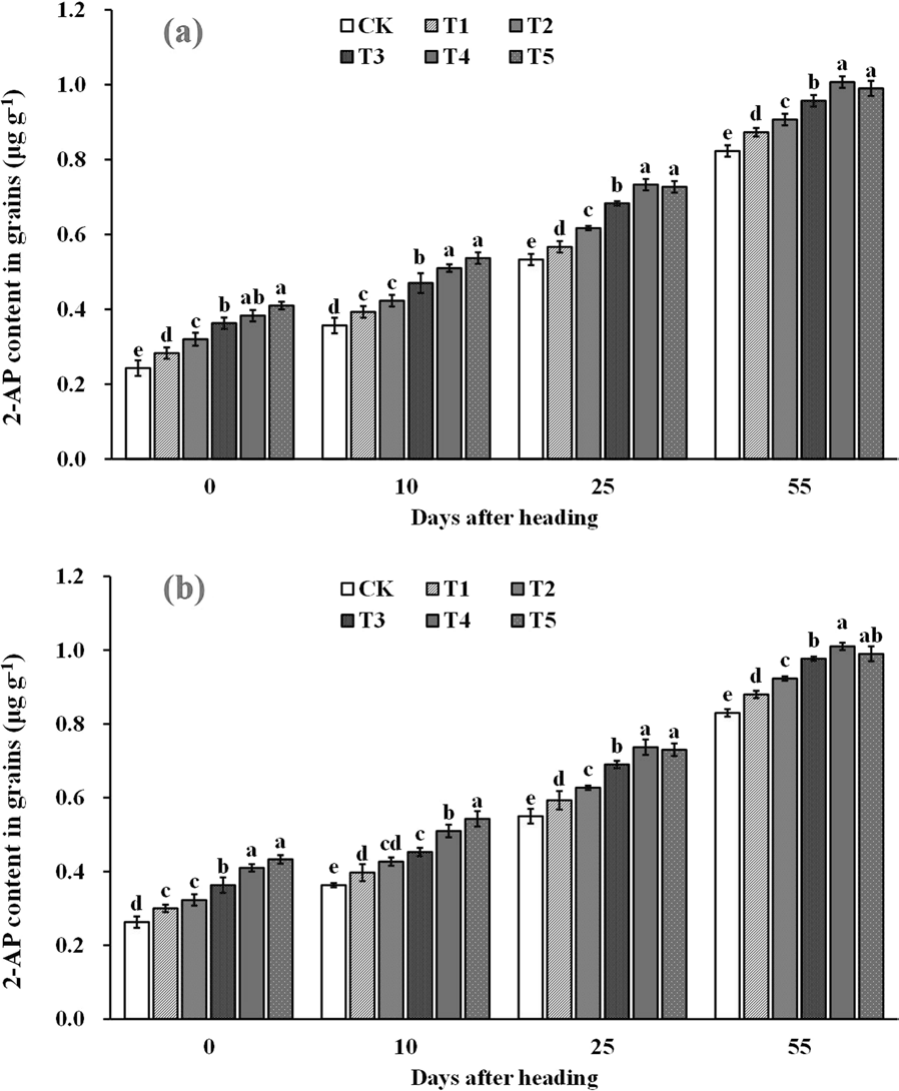
Dynamic changes of the 2-AP content in rice grain in 2019 (**a**) and 2020 (**b**). Error bars show standard error of replicates (n = 3). Values followed by different lowercase letters were significantly different at the 0.05 probability level among different treatments

The 2-AP content in the grains increased gradually during the rice aroma formation period after heading (shown in Fig. 12). It was observed that spraying ZnO NPs significantly increased the 2-AP content in the grains during all the time periods after heading. In comparison with the CK, the 2-AP content in the grains increased by 15.3–66.7%, 9.3–44.0%, 7.1–35.8%, and 6.1–21.4% at the heading stage, 10 days after heading, 25 days after heading, and maturity stage, respectively, by spraying ZnO NPs. The 2-AP content of rice showed an increasing trend with the increase in the sprayed ZnO NPs dosage from the heading stage to 10 days after heading. However, a further increase in the 2-AP content was not observed when the dosage of sprayed ZnO NPs exceeded the T4 between 25 days after heading stage and maturity stage, and a tendency of a decrease in grain 2-AP content was observed.

## Discussion

Zinc plays a crucial role in coordinating the accumulation of reactive oxygen species in plants, which is essential for their antioxidant capacity. Insufficient zinc in rice can lead to reduced chlorophyll content in crop leaves, hampering photosynthesis and ultimately impacting crop yield and quality (Chutipaijit et al. 2018; Wang and Jin 2005). Therefore, proper zinc supplementation is vital for rice production and the formation of high-quality rice (Zhang et al. 2021a). In this study, the foliar application of ZnO NPs significantly improved grain yield (2.3–4.1%) by increasing the number of spikelets per spike (7.4–9.2%), filled grain rate (1.7–4.3%), and 1000-grain weight (4.2–7.1%). This improvement might be related to the "N-Zn mutual promotion" effect during the late nutrient transport process. The application of ZnO NPs promoted nitrogen translocation within the plant, leading to an increase in grain nitrogen content (Fig. 9), enhanced grain filling, and reduced grain deterioration (Kutman et al. 2010; Xia et al. 2018; Chen et al. 2022). Furthermore, a moderate increase of nitrogen in grains at the mid stage of rice leads to a decrease in the content of straight-chain starch, improving the cooking quality (Zhang et al. 2021b). Our results showed that the foliar application of ZnO NPs at the gestation stage significantly improved the breakdown value while decreasing the setback value of rice (Table 3). Higher disintegration value and lower recovery value are associated with low hardness, high viscosity, and good elasticity, indicating that such rice tends to have a great taste (Table 4) (Yin et al. 2021; Li et al. 2020). Regarding the absorption of other micronutrients by the grains, the foliar spray of ZnO NPs at the gestation stage mainly showed an inhibitory influence on the grain absorption of Fe and Cu at medium to high dosages. This may be due to certain antagonistic interactions of zinc with both Fe and Cu (Imtiaz et al. 2003; Rai et al. 2021; Kumar et al. 2022; Saha et al. 2017).

Effective enrichment of grain zinc does not solely rely on increasing rice grain zinc content and accumulation; it also requires enhancing zinc content and bioavailability efficiency within the final rice products (Saha et al. 2017). Phytic acid, as an anti-nutritional factor, tends to chelate with metal ions and form insoluble salts, limiting the body's absorption and utilization of these elements (Perera et al. 2018). Rice bran contains a significant amount of phytic acid, leading to an uneven distribution of zinc in the rice grain, with the edible endosperm portion having lower zinc content (Saenchai et al. 2016). Therefore, the phytic acid to zinc molar ratio is often used as a measure of zinc effectiveness in rice products (Gibson et al. 2010). Khampuang et al. (2020) found that foliar spraying of zinc fertilizers at low levels could improve zinc content while reducing the phytic acid content of rice edible parts, resulting in higher zinc bioavailability in polished rice. Similar results were observed in wheat grains and raw field pea grains (Cakmak and Kutman 2017; Poblaciones and Rengel 2016). Our study also confirmed this effect. Compared to the control group (CK), rice grains treated with ZnO NPs spray showed increased zinc content and accumulation in edible parts (Fig. 2 and Fig. 4), while the phytic acid to zinc ratio of brown rice and polished rice was reduced (Fig. 5). These findings demonstrate the positive impact of ZnO NPs spray at the gestation stage in achieving effective zinc enrichment in rice grains.

2-Acetyl-1-pyrroline (2-AP), a key component responsible for rice aroma, serves as a determinant of the intensity of rice fragrance (Wakte et al. 2017). Its primary synthetic pathway often relies on proline as the substrate, involving several key steps. Initially, proline is catalyzed by proline oxidase, leading to the oxidation of pyrrole-5-carboxylic acid (P5C). Subsequently, pyrrole-5-carboxylic acid (P5C) undergoes decarboxylation via pyrrole-5-carboxylic acid decarboxylase (P5CR) to produce 1-pyrrolidine. Finally, 1-pyrroline binds with acetyl coenzyme A, provided by carbon metabolism, in the presence of pyrimidine acetyltransferase, resulting in the formation of 2-AP (Okpala et al. 2019). In our study, the increased 2-AP content in rice grains was primarily attributed to the significant enhancement in proline content and proline oxidase activity within the grains. Upon further examination of the dynamic changes in a series of physiological indices after ZnO NPs application, we observed that the fundamental reason lies in the significant improvement in enzyme activities (NRA and GSA) related to nitrogen synthesis in the leaves (Figs. 6 and 7), subsequently impacting nitrogen synthesis and transport (Figs. 8 and 9). Notably, nitrogen positively influences both proline content and proline oxidase activity in the grain (Yang et al. 2021; Mo et al. 2018). The increase in nitrogen synthase activity with ZnO NPs application may be attributed to the moderate Zn supplementation, which enhances leaf antioxidant capacity and delays leaf senescence, thereby promoting photosynthesis during the middle and late growth stages, ultimately positively affecting nitrogen synthesis (Cherif et al. 2010; Wang and Jin 2005; Takashima et al. 2004). However, further research is required to delve into the intricacies of this process and understand its underlying mechanisms.

## Conclusion

In this study, we investigated the effects of foliar application of different dosages of ZnO NPs during the gestation stage of high-quality palatable japonica rice. Our findings revealed significant advancements in zinc biofortification and aroma enhancement in rice grains. The application of ZnO NPs resulted in increased zinc content in all parts of the grains, with a notable enrichment in the edible portions, thereby enhancing the bioavailability of this essential nutrient. Moreover, ZnO NPs proved instrumental in shaping the aroma profile of rice grains. By boosting nitrogen metabolism in the flag leaves and elevating nitrogen and proline content in the grains, ZnO NPs stimulated the activity of proline oxidase, ultimately leading to a substantial increase in 2-acetyl-1-pyrroline (2-AP) content. Our comprehensive analysis indicates that foliar spraying of ZnO NPs during the gestation stage effectively enhances both zinc biofortification and aroma in rice grains. Optimal results were achieved at a spraying rate of 3.0–6.0 kg hm^−2^, considering the balance between yield and rice quality. These findings hold tremendous potential for improving the nutritional value and sensory experience of rice. The application of ZnO NPs as a foliar zinc fertilizer offers a promising avenue for enhancing human health and satisfaction through fortified zinc levels and captivating rice aroma.

## Data Availability

Data will be made available on request.

## Abbreviations

Zn: Zinc
ZnO NPs: Zinc oxide nanoparticles
NRA: Nitrate reductase activity
GSA: Glutamine synthetase activity
